# A graphene based frequency quadrupler

**DOI:** 10.1038/srep46605

**Published:** 2017-04-18

**Authors:** Chuantong Cheng, Beiju Huang, Xurui Mao, Zanyun Zhang, Zan Zhang, Zhaoxin Geng, Ping Xue, Hongda Chen

**Affiliations:** 1 State Key Laboratory of Integrated Optoelectronics, Institute of Semiconductor, Chinese Academy of Sciences, Beijing, 100083, PR China; 2 State Key Laboratory of Low-Dimensional Quantum Physics and Center for Atomic and Molecular Nanoscience, Department of Physics, Tsinghua University, Beijing, 10084, PR China; 3 College of Materials Science and Opto-Electronic Technology, University of Chinese Academy of Sciences, Beijing, 100049, PR China; 4 School of Electronics and Information Engineering, Tianjin Polytechnic University, Tianjin 300387, PR China; 5 School of Electronic and Control Engineering, Chang’an University, Xi’an 710064, PR China; 6 Collaborative Innovation Center of Quantum Matter, Beijing, 100084, PR China

## Abstract

Benefit from exceptional electrical transport properties, graphene receives worldwide attentions, especially in the domain of high frequency electronics. Due to absence of effective bandgap causing off-state the device, graphene material is extraordinarily suitable for analog circuits rather than digital applications. With this unique ambipolar behavior, graphene can be exploited and utilized to achieve high performance for frequency multipliers. Here, dual-gated graphene field-effect transistors have been firstly used to achieve frequency quadrupling. Two Dirac points in the transfer curves of the designed GFETs can be observed by tuning top-gate voltages, which is essential to generate the fourth harmonic. By applying 200 kHz sinusoid input, arround 50% of the output signal radio frequency power is concentrated at the desired frequency of 800 kHz. Additionally, in suitable operation areas, our devices can work as high performance frequency doublers and frequency triplers. Considered both simple device structure and potential superhigh carrier mobility of graphene material, graphene-based frequency quadruplers may have lots of superiorities in regards to ultrahigh frequency electronic applications in near future. Moreover, versatility of carbon material system is far-reaching for realization of complementary metal-oxide-semiconductor compatible electrically active devices.

Frequency multipliers, cooperated with low frequency local oscillators, can provide high frequency electromagnetic signal for radio astronomy, radar technology and other communication applications^1^,^2^,^3^. Frequency multipliers with high multiplication factors can release required operation frequency of local oscillator systems, which results in an easily available high frequency generator. However, in conventional frequency multipliers, output powers of high-order harmonics are much lower than low-order harmonics. Therefore, high-multiplication frequency multipliers are often achieved by cascading multi-stage frequency doublers^4^,^5^,^6^. However, this kind of high-multiplication frequency multiplier is complex in the structure and usually costly.

On the other side, graphene, the first discovered two-dimension monolayer material^7^, has aroused extensive research interests due to its outstanding electronic^7^,^8^, optical^9^,^10^, mechanical^11^, and thermal properties^12^. Combining its ultra-high electron mobility, tunable electronic transport polarity and ultrastrong intrinsic strength, graphene functions as a promising candidate for solid electronic^13^,^14^,^15^,^16^, flexible electronic^17^,^18^,^19^, and sensing^20^,^21^,^22^,^23^. Graphene has wide applications for future high frequency electronics because of its ultrahigh carrier mobility and saturation velocity^8^. With the unique ambipolar behavior, graphene field effect transistor (GFET) based frequency doublers^24^,^25^, frequency triplers^26^, mixers^27^,^28^, and demodulators^29^ have been accomplished. Due to linear dispersion relation of energy band of graphene^30^, Fermi level of graphene can be easily shifted across the Dirac cone from valence band to conduction band or reversely by applying a tunable external electrostatic field, thus resulting in the V-shaped transfer characteristic (I_ds_–V_gs_) curves of GFETs^31^. With this new type non-linear transfer characteristic, several groups have achieved frequency doublers with high output signal purity (>90%) with just a single GFET and without any additional filters^24^,^25^,^32^. Chen *et al*. developed the new concept and reported the first graphene-based frequency tripler constructed by two GFETs connected in series^26^. By separately tuning the doping levels of graphene in the two GFETs with an external electrostatic field, a W-shaped transfer characteristic was obtained, which is critical for high spectral purity (>70%) frequency triplers^26^. However, one GFET is enough for the purpose of frequency tripling. We have reported a pure frequency tripler with ultrahigh output signal purity (>94%) with only one GFET for the first time^33^. The CVD grown graphene with micron scale graphene flakes interspersed on the surface was used as the channel material of the GFET. A W-shaped transfer curve was obtained due to different doping levels of the single layer graphene and the bilayer graphene in the channel. In order to achieve a high performance frequency quadrupler with our reported device structures, lots of work optimizing synthesis conditions would be required to achieve a more perfect W-shaped transfer curve with two symmetrical Dirac points. In this work, a much simpler method was carried out to realize a tunable W-shaped transfer curve with two Dirac points. More specifically, a dual-gated GFET with a W-shaped transfer curve was firstly used to achieve frequency quadrupling. With an input of 200 kHz sinusoid signal, about half of the output signal radio frequency (RF) power is concentrated at desired 800 kHz. Moreover, in suitable operation areas, our devices can also work as high performance frequency doublers and frequency triplers, respectively. Compared with the first reported graphene-based frequency tripler^26^, this work shows a remarkable improvement in operation bandwidth with a relative simple device configuration. The simple device structure and potential superhigh carrier mobility of graphene make this graphene-based frequency quadrupler prospective for ultrahigh-frequency electronic applications in the future. Complementary metal-oxide-semiconductor (CMOS) compatible fabrication processes give these carbon material-based devices a chance to replace traditional silicon material in analog circuit, especially in RF applications.

## Results and Discussion

### Structure design and device fabrication

The V-shaped transfer curves can be obtained easily in GFETs if homogeneous graphene material is used as the channel material^7^. Total channel resistance (R_total_) can be written as^34^:


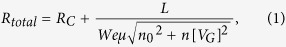


where R_C_ is the metal/graphene contact resistance (nearly a constant)^35^, W and L are the width and the length of the channel, respectively, μ is the mobility of the charge carrier, n_0_ is the electron-hole puddle^36^ concentration (a constant), e is the electron charge, and n is gate (V_G_) dependent charge carrier concentration which can be described by:


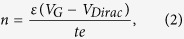


where, ε and t are the permittivity and thickness of the dielectric layer and V_Dirac_ is the gate voltage of charge neutrality point. Hence n equals to zero with V_G_ reaching V_Dirac_, and the minimal drain current of the V-shaped transfer curve can be obtained as R_total_ reaches the peak value. However, if inhomogeneous graphene, such as a graphene PN junction^37^, is used in the FET channel, then equation (1) will be replaced by:


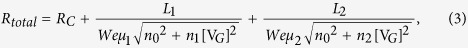


as two graphene parts are connected in series. Here, L_1_ and L_2_ are the length of the N-type doping graphene and P-type doping graphene, respectively. μ_1_ and μ_2_ are the carrier mobility in the two regions. As equation (2), the charge carrier concentrations in the two different regions can be given by:


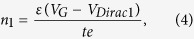



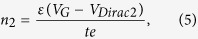


where V_Dirac1_ and V_Dirac2_ are the Dirac voltage of N-type region and P-type region, respectively. With different values of V_Dirac1_ and V_Dirac2_, two maxima will be discovered in R_total_ at gate voltages of V_Dirac1_ and V_Dirac2_.

**Figure 1:**
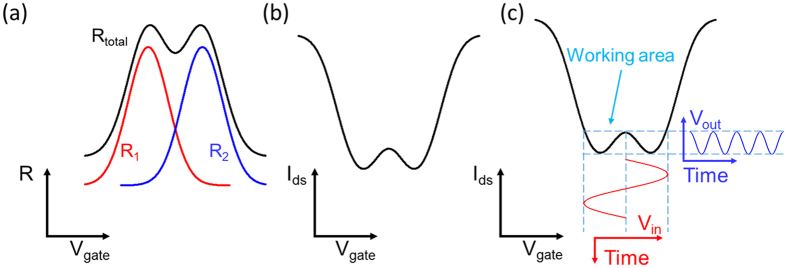
Sketch describing the operation principle of the graphene based frequency quadruplers. (**a**) Both resistances of graphene 1(R_1_) and graphene 2 (R_2_) with different doping levels and the total channel resistance of them connected in series (R_total_) are as a function of gate voltage. (**b**) The corresponding transfer curve is shown as the drain current is inversely proportional to total channel resistance. (**c**) Generation of four-cycle waveform at V_out_ from one-cycle input V_in_ results from relationship between I_ds_ and V_gate_ in the working area.

Figure 1a and b sketchily display formation of the W-shaped transfer curve in a single GFET with two different graphene parts connected in series as the channel material. In Fig. 1a, due to different doping levels, gate voltages for resistance maxima in graphene 1 (red line) and graphene 2 (blue line) are different. The black line with two resistance maxima is the total channel resistance of GFET. Since the drain current is inversely proportional to the total channel resistance, a W-shaped transfer curve with two conductance minima can be obtained, which is shown in Fig. 1b. Figure 1c shows how GFETs with W-shaped transfer curves can work as frequency quadruplers. The device can generate four-cycle waveform at V_out_ with one-cycle input V_in_. Therefore, a frequency quadrupler can be realized with only one GFET if two different graphene parts with different doping levels, such as graphene PN junctions, are formed in the channel.

Although no effective bandgap, graphene-based PN junctions find their applications in electronic^38^,^39^ and optoelectronic fields^40^,^41^. Stable graphene PN junctions can be obtained by electrostatic doping^42^,^43^, chemical doping^44^,^45^, substrate engineering^46^ and photoinduced doping^47^,^48^. Among these methods, electrostatic doping method is tunable in real time by applying variable gate voltages^42^,^43^. In our case, a tunable doping method is aspired for the purpose of achieving a tunable W-shaped transfer curve. Therefore, a narrow top gate (TG) is utilized to dope the underneath graphene with electrostatic field and form PN junctions in the graphene channel of GFET. A dual-gated GFET is designed and fabricated to achieve frequency quadrupling function for the first time since the dual-gated GFET structure can modulate charge transport characteristics in graphene films efficiently^34^. In this case, the total channel resistance can be derived as:


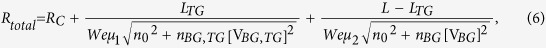


where L is the channel length, L_TG_ is the length of the TG, and the charge carrier concentrations in graphene under the TG and away from the TG can be given by:


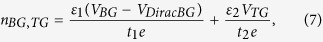



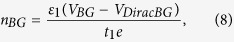


where ε_1_, ε_2_ and t_1_, t_2_ are the permittivities and thicknesses of the back gate (BG) dielectric layer and TG dielectric layer, respectively, V_Diarc BG_ is the Dirac voltage of the channel graphene without TG and V_BG_ is the BG voltage. The peak value of R_total_ can be obtained by setting either equation (7) or equation (8) to zero. Therefore, a W-shaped transfer curve with two Dirac points can be achieved with this dual-gated GFET structure. The values of the two Dirac voltages can be obtained from equation (7) and equation (8), respectively, which are shown as:


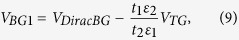



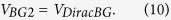


Hence the newly arising Dirac voltage V_BG1_ is tunable with variable TG voltages and a linear relation can be observed from equation (9).

**Figure 2:**
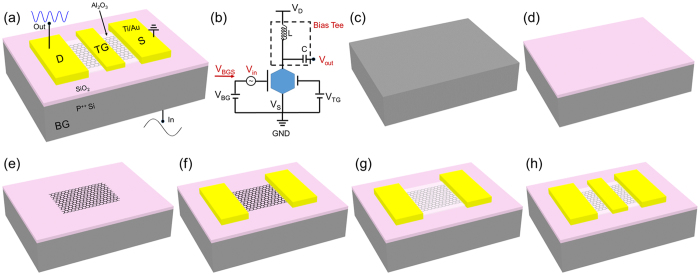
A dual-gated GFET-based frequency quadrupler. (**a**) The three-dimensional layout of the basic device structure. The P^++^ silicon with the SiO_2_ cover layer is working as global BG and the narrow Ti/Au finger with the Al_2_O_3_ bottom layer is utilized as local TG. A one-cycle input electronic signal at BG can result in a four-cycle output signal at the drain electrode. (**b**) Circuit schematic used in this work to actualize the dual-gated GFET-based frequency quadruplers. A high performance bias tee was used to apply DC bias and extract output RF power. (**c**) The device began from a heavily doped silicon wafer. (**d**) A SiO_2_ layer with thickness of 100 nm was completed by a dry thermal oxidization process. (**e**) Wet-transfer of CVD grown graphene onto the surface of the SiO_2_ layer. The patterned graphene was defined by lithography and O_2_ plasma etching. (**f**) With photolithography, thermal evaporated and lift-off processes, Au/Ti pads working as contact electrodes were obtained. (**g**) Al_2_O_3_ layer with thickness of 40 nm was achieved on graphene with an ALD technique. Before this process, a 1 nm Al seed layer was deposited on graphene with electron beam evaporation. (**h**) The device was completed with fabrication of the Ti/Au finger working as local TG in the center of the GFET channel.

The three-dimensional schematic of the dual-gated GFET-based frequency quadrupler is shown in Fig. 2a. The P^++^ silicon with the SiO_2_ cover layer works as global BG. The narrow Ti/Au finger with Al_2_O_3_ bottom layer is utilized as local TG. With suitable TG voltage, the one-cycle input electronic signals applying to global BG can result in the four-cycle output signals at the drain electrode. The circuit implementation of the frequency quadrupler is displayed in Fig. 2b. A common-source topology is used for dual-gated GFET in this frequency quadrupler. At first, a suitable voltage V_TG_ is applied to the TG to achieve electrostatic doping in the underneath graphene. Direct-current (DC) voltage V_BG_ is applied to BG to bias the working point of the frequency quadrupler, while an alternating current (AC) sinusoidal input signal V_in_ is added to V_BG_. V_S_ is connected to the ground. High performance bias tee is utilized to apply DC bias and extract the output RF power.

The detailed device fabrication process of dual-gated GFETs is schematically illustrated in Fig. 2c–h. A heavily doped silicon chip was used as the substrate and global BG (Fig. 2c). Then the silicon wafer was put into an atmospheric thermal oxidation furnace with a temperature of 1000 °C for 3 h. A 100 nm-thick SiO_2_ layer working as the dielectric layer of BG was formed on the surface of the silicon substrate (Fig. 2d). Then the commercial CVD grown graphene film was transferred onto the surface of the thermal oxidization SiO_2_ layer after the copper foil being etched away. The photolithography and O_2_ plasma etching were successively used to fabricate the patterned graphene channel (50 μm * 20 μm) that works as the active region (Fig. 2e). The Ti (10 nm)/Au (200 nm) films working as metal contacts were competed by lift-off in acetone after the photolithography and thermal evaporated processes (Fig. 2f). In order to obtain the dielectric layer for TG, 40 nm Al_2_O_3_ dielectric was generated on the graphene films successfully by atomic layer deposition (ALD) (Fig. 2g). Here, it should be noted that before the ALD process, a 1 nm Al seed layer was deposited on the graphene surface with electron beam evaporation. After deposition of the Al_2_O_3_ dielectric layer, photolithography and wet etching processes were carried out to etch the Al_2_O_3_ layer on the surface of the contact pads. At last, the Ti (10 nm)/Au (200 nm) films working as TG were finished by lift-off in acetone after the photolithography and thermal evaporated processes (Fig. 2h). As the fabrication temperatures of these processes mentioned above are lower than 200 °C, CMOS compatible fabrication process is achieved^49^.

### Characterization of the fabricated device

**Figure 3:**
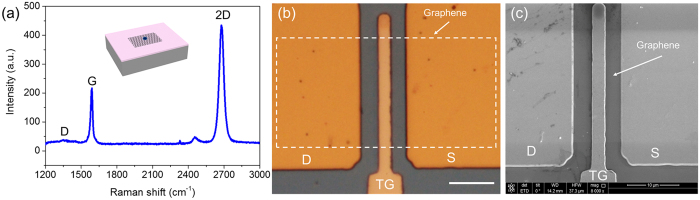
Characterization of the fabricated device. (**a**) Raman spectrum of monolayer graphene obtained at the position of the black dot in the inset. (**b**) Optical microscope image of dual-gated GFET and the white dotted frame shows the graphene location. The scale bar is 10 μm. (**c**) SEM of the device surface where the graphene boundary can be discovered due to high electrical conductivity of graphene. The length of TG is 2.3 μm.

Before deposition of the Al_2_O_3_ dielectric layer, Raman spectrum (Fig. 3a) of graphene was obtained utilizing a 532 nm excited laser with incident power of 5 mW and laser spot diameter of 20 μm. Intensity ratio of 2D/G is 3, indicating monolayer nature of the CVD graphene sample. The almost invisible D band suggests high quality of the graphene film. Figure 3b shows the optical micrograph of the fabricated dual-gated GFET-based frequency quadrupler. The channel length and width of fabricated GFET are 9.5 μm and 20 μm, respectively. The length of TG is 2.3 μm. The white dotted frame shows location of the single layer graphene. The graphene layer was visible on the surface of the 100 nm SiO_2_ layer and became invisible after deposition of 40 nm Al_2_O_3_ layer. Scanning electron microscopy (SEM) image of the device surface is displayed in Fig. 3c. The boundary of the buried graphene layer can be discovered with easiness because of high electrical conductivity of graphene.

### Static response of the device

**Figure 4:**
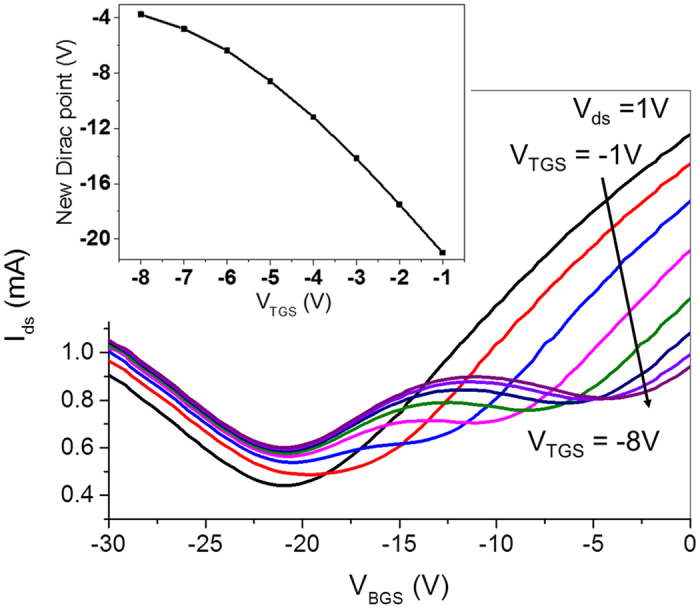
The DC transfer curves of dual-gated GFET with different TG voltages (from −1 V to −8 V) at V_ds_ of 1 V. The inset illustrates that gate voltage of the new Dirac point is approximately a linear function of V_TGS_ at low V_TGS_ and a nonlinear relationship for high V_TGS_ (absolute value).

Figure 4 shows the DC transfer curves of dual-gated GFET with different TG voltages. BG voltage V_BGS_ (V_BG_-V_S_) swept from −30 V to 0 V and bias voltage V_ds_ (V_D_-V_S_) was set at 1 V. By decreasing TG voltage V_TGS_ (V_TG_-V_S_) from −1 V to −8 V, the second obvious Dirac point is arising. When V_TGS_ was set at −1 V, only one Dirac point at about −21 V is discovered, indicating homogenization of the whole graphene channel and heavy n-type doping of the original graphene. When V_TGS_ was decreased to −8 V, two clear conductance minima can be found at the BG voltages of about −21 V and −4 V, respectively. Occurrence of the second Dirac point at −4 V indicates formation of soft n-doped graphene underneath TG. The inset shows relationship between location of the newly formed Dirac point and TG voltages and illustrates that new Dirac voltage is approximately a linear function of TG voltage, which agrees well with equation (9). With decrease of TG voltage, an increasing number of holes will be injected into the graphene layer underneath TG due to capacitance effect, which results in decrease of the doping level of graphene. As the doping level of original heavy n-type graphene away from TG in the channel remains unchanged, a constant Dirac voltage at −21 V (in agree with equation (10)) can be discovered. An interesting phenomenon is that the new Dirac point becomes obvious with decrease of V_TGS_. In other words, the total area of the soft n-doped graphene increases with decrease of V_TGS_. Therefore, resistance of this part graphene plays a growing important role in the total channel resistance. The reason is that TG can dope the graphene near the TG edges due to edge effect of the capacitor structure and the total doping range increases when V_TGS_ decreases (increase in absolute value). Nonlinear behaviour in the inset at lower V_TGS_ is another result of edge effect proposed above. Therefore, with narrow TG for underneath graphene electrostatic doping, obvious inhomogeneous graphene could be formed in the FET channel and a W-shaped transfer curve was obtained with dual-gated GFET.

### Dynamic response of the device

**Figure 5:**
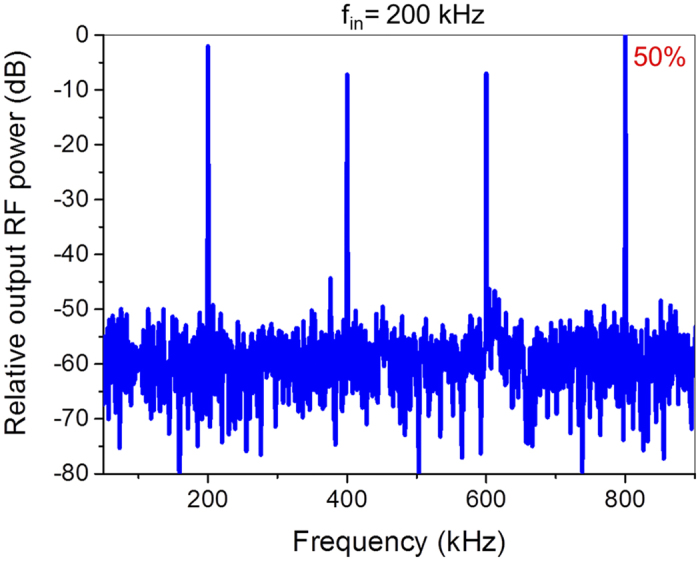
The relative output RF power spectrum for a 200 kHz input sinusoidal signal was obtained by Fourier transform of the output signal waveforms and about 50% of the output RF power is concentrated at 800 kHz.

Figure 5 shows the measured AC performances of our dual-gated GFET based frequency quadrupler. In the AC measurements, the V_TGS_ was set at −8 V and the V_ds_ was set to 1 V. The capacitor C from a bias tee was used to block the DC signal from the output V_out_. A 200 kHz sinusoidal input (V_BGS_ ranges from −22 V to −2 V) was applied to the BG to initiate the frequency quadrupling. In principle, a pure frequency quadrupler (nearly 100% spectrum purity) can be achieved when two symmetric conductance minima arise in the operation area simultaneously, which is sketchily shown in Fig. 1c. In other words, a higher symmetrical factor in the working area can result in a higher output power of the fourth harmonic. The relative output RF power spectrum is obtained through the Fourier transform of the output real time RF signals. With these data, it is found that about 50% of the output RF power is concentrated at the desired 800 kHz. Compared with the useful frequency, these undesired output frequency components (about 25% at the fundamental frequency, about 12% at the second harmonic and about 12% at the third harmonic) play a minor role. In order to investigate the RF conversion efficiency of the frequency quadrupler, the RF input power was obtained. As the total capacitance of the dual-gated GFET is about 18 pF, the total RF input power is about 1.7 mW. The quadrupled output power is 2.4 μW, which means that a RF conversion efficiency of 0.14% is achieved in this frequency quadrupler. Relative low RF power conversion efficiency is attributed to the large parasitic capacitance of the fabricated GFET. Here, noticeably for the first time a GFET based frequency quadrupler is reported. Moreover, the simple dual-gated GFET based frequency quadruplers provide perfect illustration of versatility of graphene material once again.

**Figure 6:**
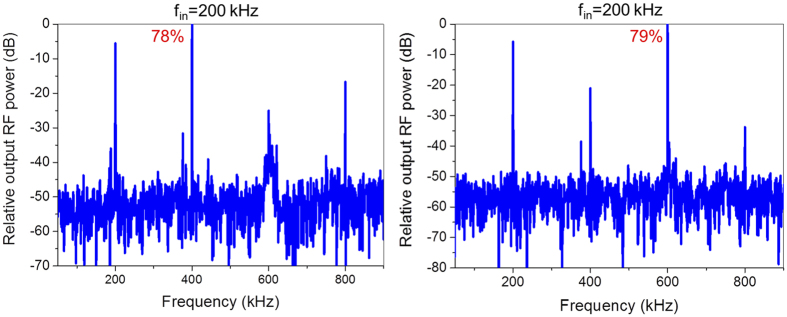
Dual-gated GFET works as high performance (**a**) frequency doublers with 78% of useful RF power and (**b**) frequency triplers with 79% of useful RF power for a 200 kHz input.

A frequency multiplier with simple structure and tunable multiplication factor may have lots of superiorities for RF electronic applications due to low cost and high integration. In our case, dual-gated GFET can also work as high performance frequency doublers or frequency triplers with a suitable operation area. Figure 6a shows relative output RF power spectrum of working dual-gated GFETs as frequency doublers (V_TGS_ = −6 V and V_BGS_ ranged from −10 V to −2 V) and about 78% of output RF power is concentrated at useful 400 kHz, with an input of 200 kHz. Figure 6b presents AC performances of working dual-gated GFETs as frequency triplers, where V_TGS_ was set to −8 V and V_BGS_ swept from −15 V to −1 V. About 79% of total RF power is concentrated at 600 kHz with 200 kHz input. Therefore, dual-gated GFET can work as high performance frequency doublers, frequency triplers and frequency quadruplers by choosing suitable operation areas. This is the first time that a multi-mode frequency multiplier is reported.

With potential ultrahigh carrier mobility and high saturation velocity, graphene can response to high frequency signals completely. Hence large RC time constant of the device structure is major obstacle to improvement of operation bandwidth of graphene based device. Therefore, performance of graphene based devices can be enhanced significantly if an optimized fabrication process is carried out to decrease both parasitic capacitance and output resistance. More clearly, in order to decrease the parasitic capacitance of the GFET-based frequency quadrupler, a highly resistive substrate such as quartz assisting with a local BG technique is preferred. On the other hand, both contact resistance and channel resistance make contribution to output resistance of GFET. A polymeric residue-free graphene fabrication process^50^ can be carried out to reduce contact resistance. Channel resistance can be decreased by fabricating GFET with nanoscale channel length and large W/L ratio. In order to investigate limitation of operation frequency of dual-gated GFET-based frequency quadrupler, a developed device structure is designed and proposed. The channel width and length of newly designed GFET are 10 μm and 1 μm, respectively. A local BG with width of 1 μm is located at the bottom of the channel. A SiO_2_ gate dielectric layer with thickness of 90 nm is formed between BG and the graphene channel. With this structure, time constant of about 1.74 ps (R of about 200 Ω and C of about 8.7 fF) can be obtained, indicating cut-off frequency of about 92 GHz and 368 GHz generated output signal. Under a 1 V bias voltage, required carrier mobility of graphene is only 3680 cm^2^/Vs, which can be achieved easily due to ultrahigh intrinsic carrier mobility of graphene material. Hence graphene-based frequency quadruplers have great potential to generate ultrahigh frequency signals and could find its roles easily in ultrahigh frequency electronic applications in near future.

Here, we summarize potential superiorities that dual-gated GFET offers as a new device for RF applications.

- A multi-mode frequency multiplier. The zero band gap of graphene enables tunable electronic transport polarity. In this work, a narrow top gate was utilized to dope underneath graphene with electrostatic field, and a tunable transfer curve was achieved with one GFET. With the tunable transfer curve, GFET can work as high performance frequency doubler, frequency tripler and frequency quadrupler.
- High multiplication factor. Without any additional filter system, it is impossible to achieve a frequency multiplier with high multiplication factor by using traditional nonlinear electronic devices because output power of the high-order harmonic is much smaller than the low-order harmonic. In this work, a graphene based frequency quadrupler with about 50% power purity was realized with dual-gated GFET. Developing idea proposed in this work, a frequency multiplier with multiplication factor of 2 * N (N > 2) can be realized by fabricating N-1 top gates in the GFET channel. With superhigh carrier mobility of graphene, this graphene-based frequency multiplier will play an important role in the ultrahigh-frequency electronic applications, such as signal generator for THz.
- Low cost. The cost-effective high performance CVD graphene material can be available with rapid improvement of the synthetic technology. Simplicity of the GFET structure results in low fabrication cost. Furthermore, the demand of this multi-mode frequency multiplier increasing rapidly as communications would become indispensable nowadays.

## Conclusion

In summary, a novel dual-gated GFET based high performance frequency quadrupler is presented. Benefit from electrostatic doping effect of the narrow TG to the underneath graphene, a W-shaped transfer curve was obtained with only one GFET. With this new type nonlinear I-V feature, a graphene-based frequency quadrupler was achieved. This device can also operate as high performance frequency doublers and frequency triplers. To the best of our knowledge, it is the first reported multi-mode graphene based frequency multiplier in the world. The potential ultrahigh carrier mobility of the graphene, together with simplicity of the device structure and CMOS compatible fabrication processes makes the dual-gated GFET based frequency quadrupler one of the most captivating candidates for future ultrahigh-frequency electronics, especially RF applications.

## Methods

We characterized the fabricated device at room temperature in ambient conditions. The DC characteristics of the dual-gated GFET were measured by a Keithley 1602B semiconductor analyzer. Both Agilent 33250A signal generator and Agilent MSO-X 3034A mixed signal oscilloscope were used to obtain AC characteristics of the graphene based frequency quadrupler.

## Additional Information

**How to cite this article:** Cheng, C. *et al*. A graphene based frequency quadrupler. *Sci. Rep.* **7**, 46605; doi: 10.1038/srep46605 (2017).

**Publisher's note:** Springer Nature remains neutral with regard to jurisdictional claims in published maps and institutional affiliations.

## References

[b1] Raisanen A. V. Frequency multipliers for millimeter and submillimeter wavelengths. Proc. IEEE 80, 1842–1852 (1992).

[b2] Riley J. R. & Smith A. D. Design considerations for an harmonic radar to investigate the flight of insects at low altitude. Comput. Electron. Agricult. 35, 151–169 (2002).

[b3] Chien G. & Gray P. R. A 900-MHz local oscillator using a DLL-based frequency multiplier technique for PCS applications. IEEE J. Solid-State Circuits 35, 1996–1999 (2001).

[b4] Bao M. Q., Kozhuharov R., Chen J. J. & Zirath H. A D-band keyable high efficiency frequency quadrupler. IEEE Microw. Wireless Compon. Lett. 24, 793–795 (2014).

[b5] Chen G. Y. . Design and analysis of Ka -band monolithic high-efficiency frequency quadrupler using GaAs HBT–HEMT common-base/common-source balanced topology. IEEE Trans. Microw. Theory Tech. 61, 3674–3689 (2013).

[b6] Abbasi M. & Ricketts D. S. 275–285 GHz balanced frequency quadrupler chain in 45 nm SOI CMOS. Electron. Lett. 51, 1424–1425 (2015).

[b7] Novoselov K. S. . Electric field effect in atomically thin carbon films. Science 306, 666–669 (2004). 15499015 10.1126/science.1102896

[b8] Bolotin K. I. . Ultrahigh electron mobility in suspended graphene. Solid State Commun. 146. 351–355 (2008).

[b9] Nair R. R. . Fine structure constant defines visual transparency of graphene. Science 320, 1308 (2008). 18388259 10.1126/science.1156965

[b10] Wang F. . Gate-variable optical transitions in graphene. Science 320, 206–209 (2008). 18339901 10.1126/science.1152793

[b11] Lee C. G., Wei X. D., Kysar J. W. & Hone J. Measurement of the elastic properties and intrinsic strength of monolayer graphene. Science 321, 385–388 (2008). 18635798 10.1126/science.1157996

[b12] Balandin A. A. . Superior thermal conductivity of single-layer graphene. Nano Lett. 8, 902–907 (2008). 18284217 10.1021/nl0731872

[b13] Schwierz F. Graphene transistors. Nat. nanotech. 5, 487–496 (2010). 10.1038/nnano.2010.89 20512128

[b14] Lv H. M. . Inverted process for graphene integrated circuits fabrication. Nanoscale 6, 5826–5830 (2014). 24745037 10.1039/c3nr06904d

[b15] Han S. J., Garcia A. V., Oida S., K. Jenkins A. & Haensch W. Graphene radio frequency receiver integrated circuit. Nat. Commun. 5, 3086–3091 (2014). 24477203 10.1038/ncomms4086

[b16] Lin Y. M. . Wafer-scale graphene integrated circuit. Science 332, 1294–1297 (2011). 21659599 10.1126/science.1204428

[b17] Yeh C. H. . Gigahertz flexible graphene transistors for microwave integrated circuits. ACS Nano 8, 7663–7670 (2014). 25062282 10.1021/nn5036087

[b18] Tao L. Q. . Fabrication techniques and applications of flexible graphene-based electronic devices. J. Semicond. 37, 041001 (2016).

[b19] Kim K. S. . Large-scale pattern growth of graphene films for stretchable transparent electrodes. Nature 457, 706–710 (2009). 19145232 10.1038/nature07719

[b20] Shao Y. Y. . Graphene based electrochemical sensors and biosensors: a review. Electroanalysis 22, 1027–1036 (2010).

[b21] Fu W. Y. . Electrolyte-gated graphene ambipolar frequency multipliers for biochemical sensing. Nano Lett. 16, 2295–2300 (2016). 26928906 10.1021/acs.nanolett.5b04729

[b22] Lee H. . A graphene-based electrochemical device with thermoresponsive microneedles for diabetes monitoring and therapy. Nat. Nanotech. 11, 566–574 (2016). 10.1038/nnano.2016.38 26999482

[b23] Smith A. D. . Resistive graphene humidity sensors with rapid and direct electrical readout. Nanoscale 7, 19099–19109 (2015). 26523705 10.1039/c5nr06038a PMC4653760

[b24] Wang H., Nezich D., Kong J. & Palacios T. Graphene frequency multipliers. IEEE Electron Device Lett. 30, 547–549 (2009).

[b25] Ramón M. E. . Three-gigahertz graphene frequency doubler on quartz operating beyond the transit frequency. IEEE Trans. Nanotechnol. 11, 877–883 (2012).

[b26] Chen H. Y. & Appenzeller J. Graphene-based frequency tripler. Nano Lett. 12, 2067–2070 (2012). 22452648 10.1021/nl300230k

[b27] Wang H., Hsu A., Wu J., Kong J. & Palacios T. Graphene-based ambipolar RF mixers. IEEE Electron Device Lett. 31, 906–908 (2010).

[b28] Habibpour O., Cherednichenko S., Vukusic J., Yhland K. & Stake J. A subharmonic graphene FET mixer. IEEE Electron Device Lett. 33, 71–73 (2012).

[b29] Habibpour O. . Graphene FET gigabit ON–OFF keying demodulator at 96 GHz. IEEE Electron Device Lett. 37, 333–336 (2016).

[b30] McClure J. W. Band structure of graphite and de Haas-van alphen effect. Phys. Rev. 108, 612–618 (1957).

[b31] Neto A. H. C., Guinea F., Peres N. M. R., Novoselov K. S. & Geim A. K. The electronic properties of graphene. Rev. Mod. Phys. 81, 109–162 (2009).

[b32] Wang Z. . A high performance top-gate graphene field-effect transistor based frequency doubler. Appl. Phys. Lett. 96, 173104 (2010).

[b33] Cheng C. T. . A pure frequency tripler based on CVD graphene. IEEE Electron Device Lett. 37, 785–788 (2016).

[b34] Kim S. . Realization of a high mobility dual-gated graphene field-effect transistor with Al_2_O_3_ dielectric. Appl. Phy. Lett. 94, 062107 (2009).

[b35] Russo S., Craciun M. F., Yamamoto M., Morpurgo A. F. & Tarucha S. Contact resistance in graphene-based devices. Physica E 42, 677–679 (2010).

[b36] Martin J. . Observation of electron–hole puddles in graphene using a scanning single-electron transistor. Nat. Phys. 4, 144–148 (2008).

[b37] Feng T. T. . Back-gate graphene field-effect transistors with double conductance minima. Carbon 79, 363–368 (2014).

[b38] Cheianov V. V., Fal’ko V. & Altshuler B. L. The focusing of electron flow and a Veselago lens in graphene p-n junctions. Science 315, 1252–1255 (2007). 17332407 10.1126/science.1138020

[b39] Stander N., Huard B. & Gordon D. G. Evidence for klein tunneling in graphene p-n junctions. Phys. Rev. Lett. 102, 026807 (2009). 19257307 10.1103/PhysRevLett.102.026807

[b40] Liu N. . Large-area, transparent, and flexible infrared photodetector fabricated using P-N junctions formed by N-doping chemical vapor deposition grown graphene. Nano Lett. 14, 3702–3708 (2014). 24927382 10.1021/nl500443j

[b41] Lemme M. C. . Gate-activated photoresponse in a graphene p-n junction. Nano Lett. 11, 4134–4137 (2011). 21879753 10.1021/nl2019068

[b42] Özyilmaz B. . Electronic transport and quantum Hall effect in bipolar graphene p-n-p junctions. Phys. Rev. Lett. 99, 166804 (2007). 17995279 10.1103/PhysRevLett.99.166804

[b43] Choi J. H. . Complete gate control of supercurrent in graphene p-n junctions. Nat. Commun. 4, 2525–2534 (2013). 24056682 10.1038/ncomms3525

[b44] Lohmann T., Klitzing K. V. & Smet J. H. Four-terminal magneto-transport in graphene pn junctions created by spatially selective doping. Nano Lett. 9, 1973–1979 (2009). 19361173 10.1021/nl900203n

[b45] Kim S. . Graphene p–n vertical tunneling diodes. ACS Nano 7, 5168–5174 (2013). 23692508 10.1021/nn400899v

[b46] Chiu H. Y., Perebeinos V., Lin Y. M. & Avouris P. Controllable p-n junction formation in monolayer graphene using electrostatic substrate engineering. Nano Lett. 10, 4634–4639 (2010). 20886859 10.1021/nl102756r

[b47] Seo B. H., Youn J. M. & Shim M. Direct laser writing of air-stable p-n junctions in graphene. ACS Nano 8, 8831–8836 (2014). 25075554 10.1021/nn503574p

[b48] Ju L. . Photoinduced doping in heterostructures of graphene and boron nitride. Nat. Nanotechnol. 9, 348–352 (2014). 24727687 10.1038/nnano.2014.60

[b49] Chaisakul P. . Integrated germanium optical interconnects on silicon substrates. Nat. Photon. 8, 482–488 (2014).

[b50] Wu Y. . 200 GHz maximum oscillation frequency in CVD graphene radio frequency transistors. ACS Appl. Mater. Interfaces 8, 25645–25649 (2016). 27640732 10.1021/acsami.6b05791

